# Blasting Off on an Actin Comet Tail

**DOI:** 10.1371/journal.pbio.1000195

**Published:** 2009-09-22

**Authors:** Caitlin Sedwick

**Affiliations:** Freelance Science Writer, San Diego, California, United States of America

**Figure pbio-1000195-g001:**
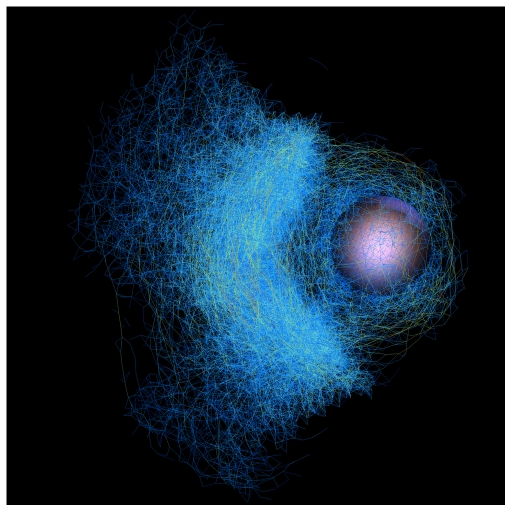
A 3-D view of the simulated actin network just as the bead breaks free from its shell and begins moving off on its comet tail (links colored by strain).

[Fig pbio-1000195-g001]Cells have structure and the ability to move, thanks in part to the actin cytoskeleton, a dynamic cellular scaffolding that facilitates many cellular processes. Actin is mostly concentrated near the outer membrane of the cell, where it is involved in such processes as the formation of membrane protrusions at the leading edges of migrating cells, the movement of certain intracellular vesicles such as endosomes, and the generation of the cleavage furrow during cell division. In this issue of *PLoS Biology*, Mark Dayel and colleagues combine computer modeling with in vitro experiments to explain some of the fundamental dynamics of actin polymerization that underlie these diverse phenomena.

The structures formed by the actin cytoskeleton are produced through the constant reshaping of chains of actin molecules. These filaments grow as free actin molecules are added to the end of the chain until actin capping proteins terminate the process. Arp2/3 can also initiate new filament growth off the sides of existing filaments to create an elaborate tree-like structure. When multiple filaments grow off the same surface, their network of branches can tangle as they grow like a stand of blackberry brambles.

As an actin network grows, it can push other objects around so that endosomes and certain intracellular pathogens appear to jet about inside cells on a “comet tail” of actin. This phenomenon can be reproduced in vitro using spherical plastic beads coated with the bacterial protein ActA (which potentiates Arp2/3 activity); when added to a solution of free actin, Arp2/3, and capping proteins, the beads grow comet tails. These beads first accumulate a thick, symmetrical shell of actin but eventually burst out of the shell, breaking its symmetry. Then, they assemble actin filaments in a directional manner and zoom away, propelled by a comet tail. The beads can move smoothly or in sporadic pulses.

In their investigation of these symmetry-breaking and motility behaviors, the authors found that they could accurately represent actin polymerization behaviors by modeling just the elastic properties of a growing actin network. They did this by modeling the actin filament network as a series of nodes connected by springs. New nodes are continually added at the surface of the bead, where ActA and Arp2/3 are growing new filaments and branches in vitro. In the model, springs attach nodes to each other, simulating the way actin trees entangle with one another in vitro. These springs exhibit resistance to compression, much like actin trees that can press together to only a limited extent. The springs also resist tension, and, just as the actin trees can rip apart from one another under tension, the springs break if pulled too much. The authors demonstrate that their in silico actin network grows as a symmetrical shell that spontaneously breaks symmetry and produces directional motility just like the in vitro actin network, showing that the elastic network properties alone are enough to explain these behaviors.

The authors' model suggests that compression plays a key role in changing the mechanical properties of the network as the shell forms. The growing actin shell forms a layer that has sparse entanglements between different actin trees. This first layer expands like an inflating balloon as actin trees grow beneath it at the bead's surface. But, as it expands, it also tightly compresses the actin trees growing beneath it, increasing their entanglements. Eventually, these inner trees get so entangled that they become brittle; to relieve the pressure, the entanglements of several actin trees rip apart at one spot, causing the shell to retract from the bead.

The authors performed simulations to investigate what happens after the actin shell breaks, and found that it opens up in two halves like a clamshell, with a linear crack and a hinge. In vitro, the shells seemed to lack this hinge, but the beads had been tightly constrained between a slide and a coverslip during the experiment. When the simulated network was similarly constrained, the hinge disappeared. And importantly, in in vitro experiments without the constraint, actin shells showed the same hinge and clamshell opening originally seen in simulations, showing that the model could predict new behavior in the experimental system.

The simulations also suggest why the bead moves smoothly under some circumstances and jerkily under others. New material added near the retracted clamshell is compressed against the shell and therefore maintains its dense interconnections, even as it presses the bead forward. Meanwhile, new material added in the area of the original rip has fewer connections because it experiences less compression and can be easily ripped anew as actin polymerization continues. Therefore, sustained, asymmetric ripping at the front edge of the bead results in smooth forward motion. Similarly, the model can explain pulsing motion; simply increasing the number of interconnections between nodes in the model necessitates more force to break the extra connections. The accumulation of more entanglements can cause the bead to become temporarily re-ensnared in its own actin shell until enough compression is built up to rip it free again. Transitions between smooth and pulsing motion might be caused by locally varying concentrations of actin-polymerizing factors that alter this degree of entanglement.

The computer model has proven a useful framework to understand actin-based motility and can already accurately predict the qualitative behavior of beads—even non-spherical ones—in in vitro experiments. It also promises to be a platform on which to build a more elaborate model that can provide predictions about the actual levels of force involved in bead motion and about the precise timing of events.

**Dayel MJ, Akin O, Landeryou M, Risca V, Mogilner A, et al. (2009) In Silico Reconstitution of Actin-Based Symmetry Breaking and Motility. doi:10.1371/journal.pbio.1000201**

